# Orthorexia Profiles in Athletes: A Multidimensional Analysis Using the Eating Habits Questionnaire (EHQ) and the Teruel Orthorexia Scale (TOS)

**DOI:** 10.3390/nu17243814

**Published:** 2025-12-05

**Authors:** María Manzanares-Cabrera, María Dolores Onieva-Zafra, Alberto Bermejo-Cantarero, Raúl Expósito-González, Daniel Lerma-García, María Laura Parra-Fernández

**Affiliations:** 1 Hospital Quiron Health Ciudad Real, 13002 Ciudad Real, Spain; maria_manz92@hotmail.com; 2 Department of Nursing, Physiotherapy and Occupational Therapy, Ciudad Real Faculty of Nursing, University of Castilla-La Mancha, 13071 Ciudad Real, Spain; mariadolores.onieva@uclm.es (M.D.O.-Z.); raul.egonzalez@uclm.es (R.E.-G.); marialaura.parra@uclm.es (M.L.P.-F.); 3 Instituto de Investigación Sanitaria de Castilla-La Mancha (IDISCAM), Castilla-La Mancha, 45004 Toledo, Spain; 4 Centro de Estudios Sociosanitarios (CESS), University of Castilla-La Mancha, 13071 Cuenca, Spain; 5 Department of Nursing, University of Illes Balears, Ibiza, Baleares, 07800 Ibiza, Spain; dani.lerma@uib.es

**Keywords:** orthorexia nervosa, healthy orthorexia, young adults, sports, dietary patterns, supplements, cluster analysis

## Abstract

**Background:** Orthorexia nervosa (OrNe) and healthy orthorexia (HeOr) are two distinct but related dimensions of interest in eating behavior research. Evidence regarding their associations with sociodemographic, dietary, and sport-related variables in physically active young adults remains limited. **Methods:** A cross-sectional study was conducted in 190 physically active young adults (53.2% women; mean age = 23.16 ± 5.13 years). Participants practiced a variety of sports including fitness (25.3%), soccer (13.7%), handball (10.5%), athletics, martial arts, cycling, and other individual or team sports. Although all participants belonged to organized sports teams or structured training groups, 38.9% were not actively competing at the time of data collection. Participants completed validated instruments assessing OrNe, HeOr, and eating-related cognitions, alongside questionnaires on sociodemographic data, dietary habits, sport discipline, training frequency, and supplement use. Hierarchical and K-means clustering were applied using the standardized scores of HeOr, OrNe, and the EHQ total score. Group differences were assessed using *t*-tests and ANOVA with effect sizes (η^2^p) reported. **Results:** Age correlated positively with OrNe, HeOr, and eating-related cognitions, indicating greater consolidation of rigid eating patterns in young adulthood. BMI was associated with OrNe only among men. Vegetarian participants showed higher nutritional knowledge but lower overall orthorexia scores. Supplement users in fitness-related sports reported higher OrNe, whereas participants in collective sports reported lower scores. Three distinct orthorexia profiles were identified, characterized by lower, slightly above-average, and higher scores on orthorexia-related variables. Participants in the higher-scoring profile showed significantly higher EHQ total, OrNe, and HeOr scores compared with the other groups (η^2^p range = 0.11–0.19). Correlations among orthorexia dimensions were positive and moderate to large. Differences between clusters in sport modality, training frequency, and supplement use underscored the influence of the sporting context. **Conclusions:** Orthorexia in young physically active adults reflects heterogeneous patterns shaped by the interplay of individual (age, sex, BMI), dietary, and sport-related factors. The identification of differentiated profiles reinforces the multidimensional nature of orthorexia and underscores the relevance of considering specific sport environments when interpreting orthorexic tendencies. Longitudinal research is warranted to examine the stability or variability of these patterns over time and to enable the use of more robust multivariate approaches that further clarify the characterization of orthorexia.

## 1. Introduction

The pursuit of healthy eating has become a growing concern in contemporary society, especially in contexts where nutrition directly influences physical performance, as in competitive athletics. While adopting healthy dietary habits can be beneficial, this concern can transform into a pathological obsession known as orthorexia nervosa (ON) [1,2]. This is characterized by an excessive fixation on consuming foods considered “pure” or “healthy,” which can lead to extreme dietary restrictions, malnutrition, and impaired quality of life [3].

Although ON is not formally recognized as an independent disorder in the Psychiatric Diagnostic Manual, such as the DSM-5 [4], it has sparked increasing interest, particularly in populations oriented toward physical performance. Importantly, ON is conceptually distinct from eating disorders driven by body image or weight-control concerns. In line with the consensus definition [5], ON is primarily motivated by the pursuit of food quality and purity rather than aesthetics or body weight. Nevertheless, certain sport environments may still facilitate the emergence of rigid purity-based eating rules. Disciplines involving strict performance routines, perfectionism, or intensive dietary monitoring may indirectly contribute to orthorexic tendencies by promoting highly controlled dietary behaviors tied to performance outcomes. In this sense, athletes who engage in high-performance contexts remain a particularly vulnerable group, as their performance is closely linked not only to physical training but also to adherence to disciplined nutritional practices [6,7]. Recent research has emphasized that orthorexic tendencies do not develop solely from dietary motivations but are also shaped by broader lifestyle and behavioral patterns. Factors such as perfectionism, stress-related coping styles, high self-imposed performance standards, and rigid health-oriented routines have been consistently linked to orthorexia-related behaviors. Individuals who display elevated health consciousness, strong adherence to structured daily habits, or a heightened need for control may therefore be at increased vulnerability to developing maladaptive forms of healthy eating [8].

The constant pressure to achieve ideal aesthetic and physiological standards has been associated with a higher likelihood of maladaptive eating behaviors increase [9]. Several studies have indicated that factors such as the type of sport, competitive level, sex, body mass index (BMI), and type of diet followed may be related to a higher risk of ON [10,11,12]. However, the findings are inconclusive, and in many cases, research analyzes these variables in isolation, which hinders a comprehensive understanding of the disorder [13].

In this context, the use of specific psychometric tools to accurately assess both healthy and dysfunctional eating behaviors becomes relevant. Instruments such as the Eating Habits Questionnaire (EHQ) [14,15] and the Teruel Orthorexia Scale (TOS) [16] have been shown to be useful in capturing different dimensions of the orthorexia phenomenon. Several authors have pointed out that some orthorexia instruments, including the EHQ, may show partial overlap between general interest in healthy eating and more dysfunctional components, generating debate about construct boundaries and factor clarity. These discussions have motivated the development of newer tools such as the TOS, which explicitly differentiates adaptive eating interest from pathological rigidity. Although further validation is still needed, both the EHQ and the TOS are currently the most widely supported alternatives to earlier measures such as the ORTO-15 and provide adequate psychometric properties for research in sport and health contexts [17].

Importantly, the complementary use of both scales provides a more complete assessment: while the EHQ primarily evaluates behavioral and emotional difficulties associated with rigid healthy eating, the TOS differentiates between a non-pathological interest in healthy eating (HeOr) and the pathological dimension of orthorexia nervosa (OrNe). The present work adopts the current consensus view of ON as a construct independent from both clinical eating disorders and normative health-motivated eating patterns. Despite their relevance, these instruments have rarely been applied jointly in athletic populations to identify differential patterns within the orthorexia construct. Despite their relevance, these instruments have rarely been applied jointly in athletic populations to identify differential patterns within the orthorexia construct.

Studies using the TOS have consistently supported its bidimensional structure. The two factors show distinct and, in some cases, opposite patterns of association with psycho-pathological variables, indicating that Healthy Orthorexia (HeOr) is not an attenuated form of Orthorexia Nervosa (OrNe) nor a precursor of risk, but rather a separate and independent dimension within the orthorexia construct. The TOS evaluates orthorexia through these two distinct dimensions: OrNe (8 items, e.g., “If I ever eat something I consider unhealthy, I punish myself for it”) and HeOr (9 items, e.g., “I mainly eat foods that I consider healthy”). All items are rated on a four-point Likert scale (0 = “strongly disagree” to 3 = “strongly agree”), which ensures a consistent scoring structure across both dimensions [18,19].

Given the coexistence of both adaptive and maladaptive patterns of healthy eating described in the literature, as well as consistent evidence of substantial interindividual variability in orthorexic tendencies, it is theoretically plausible to expect discrete subgroups within physically active populations. This provides a clear conceptual rationale for the use of cluster analysis, which enables the identification of homogeneous groups based on distinct orthorexia-related characteristics [20,21,22]. Although this methodology has been applied only infrequently in sport-related research, it has the potential to identify profiles ranging from predominantly healthy behaviors to more maladaptive patterns thereby allowing for a more nuanced characterization of orthorexia in athletes.

Likewise, it is essential to study how sociodemographic and sports variables such as sex, BMI, type of diet, or the sport practiced, influence these orthorexia profiles, as they could act as risk or protective factors [23,24]. Based on this rationale, we hypothesized that distinct orthorexia profiles would emerge in the sample and that these profiles would differ according to sex, BMI, dietary patterns, and sport type [25].

Considering the above, the present study aimed to evaluate the presence and characteristics of ON in a sample of athletes by identifying orthorexia profiles based on the scores obtained on the EHQ and TOS questionnaires. Furthermore, it aims to analyze the influence of sex, BMI, diet, and type of sport on these profiles. This multidimensional approach seeks to contribute to the understanding of ON in a population potentially vulnerable to rigid eating behaviors and to lay the foundation for future detection, prevention, and intervention strategies tailored to the sports context.

## 2. Materials and Methods

### 2.1. Study Design and Participants

This descriptive cross-sectional study was conducted between January and February 2020. Recruitment was performed through social media profiles of sports clubs, telephone contact with club managers, and direct approaches to athletes in gyms, sports halls, and local sports fields. A non-probability convenience sampling strategy was applied.

Eligible participants were young adults aged 18–35 years who engaged in regular physical activity (≥3–4 days per week). Exclusion criteria included the presence of a chronic disease requiring a specialized diet, a diagnosed eating disorder or psychiatric condition, and lack of consent to participate. A total of 190 participants meeting the inclusion criteria were enrolled in the study (53.2% women; mean age = 23.16 ± 5.13 years).

Participation was voluntary, individual, and anonymous. No financial incentives were offered. Written informed consent was obtained from all participants after receiving complete information about the aims and procedures of the study.

The study was conducted in accordance with the Declaration of Helsinki and Spanish legislation on data protection (Organic Law 15/1999, 13 December). The protocol was approved by the Ethics Committee of the University Hospital of Ciudad Real (approval code: C-279) on 23 July 2019.

### 2.2. Instruments

#### 2.2.1. Sociodemographic and Anthropometric Data

Participants completed a structured questionnaire on age, sex, marital status, educational level, employment status, type of residence (urban/rural), smoking status, and living arrangement (alone/with others). Self-reported weight (kg) and height (cm) were collected, and body mass index (BMI; kg/m^2^) was calculated. BMI categories followed the World Health Organization cut-offs: underweight (<18.5 kg/m^2^), normal weight (18.5–24.9 kg/m^2^), overweight (25.0–29.9 kg/m^2^), and obesity (≥30.0 kg/m^2^). In addition, participants provided information on their sports and exercise habits. For this study, regular physical activity was defined as engaging in structured exercise at least 3–4 days per week, irrespective of training intensity.

#### 2.2.2. Eating Habits Questionnaire (EHQ)

The Eating Habits Questionnaire (EHQ) [14] is a 21-item self-report instrument assessing orthorexia symptomatology across three subscales: (1) Knowledge of healthy eating (5 items), (2) Problems associated with healthy eating (12 items), and (3) Positive feelings about healthy eating (4 items). Items are rated on a 4-point Likert scale (1 = not at all true, 4 = very true), with higher scores indicating stronger orthorexic tendencies. Reported internal consistency values range from α = 0.87 to 0.91, with test–retest reliability between r = 0.72 and r = 0.81. The validated Spanish version was applied in this study [15]. Cronbach’s alpha values were 0.903 for the total score, and 0.769, 0.793, and 0.746 for the Problems, Knowledge, and Feelings subscales, respectively.

#### 2.2.3. Teruel Orthorexia Scale (TOS)

The Teruel Orthorexia Scale (TOS) [16] was used to assess cognitions and attitudes toward healthy eating. It comprises 17 items rated on a 4-point Likert scale (0 = strongly disagree, 3 = strongly agree). The instrument includes two correlated factors: Healthy Orthorexia (HeOr), reflecting a non-pathological interest in healthy eating, and Orthorexia Nervosa (OrNe), reflecting the negative emotional and social consequences of rigid eating patterns. HeOr was computed as the sum of nine items (item 1, item 2, item 3, item 6, item 7, item 8, item 11, item 13, and item 15), yielding a possible score range of 0–27. OrNe was computed from eight items (item 4, item 5, item 9, item 10, item 12, item 14, item 16, and item 17), with a possible score range of 0–24. In the original validation of the TOS by Juan Ramón Barrada and María Roncero (2018) [16], the mean HeOr score was 12.52 and the mean OrNe score was 3.44 in their sample of university students.

In the original validation study, Cronbach’s alpha was 0.85 for HeOr and 0.81 for OrNe, with satisfactory fit indices: χ^2^ (103) = 453.9, CFI = 0.965, TLI = 0.954, RMSEA = 0.060. In the present study, Cronbach’s alpha was 0.905 for the total score, 0.847 for HeOr, and 0.809 for OrNe, although only the two subscales (OrNe and HeOr) were used in all statistical analyses.

### 2.3. Statistical Analysis

The distribution of each variable was assessed using graphical methods complemented by the Kolmogorov–Smirnov test. Homogeneity of variances was evaluated using Levene’s test prior to the application of *t*-tests and ANOVAs. When this assumption was violated, Welch’s correction was applied. Descriptive statistics were calculated as mean ± standard deviation (SD) for continuous variables and as absolute frequencies and percentages for categorical variables. Between-group differences in continuous variables were tested using independent samples Student’s *t*-tests, while differences in categorical variables were examined with chi-square tests. For categorical variables with low expected frequencies (e.g., the Cycling subgroup), Fisher’s Exact Test was applied instead of the χ^2^ test.

Pearson correlation coefficients were computed to examine associations between age, BMI, and scores on the TOS, the EHQ, and their respective subscales. The strength of correlations was interpreted as follows: <0.10, trivial; 0.10–0.29, small; 0.30–0.49, moderate; ≥0.50, large. Based on these coefficients, a semantic correlation network was generated using the networkx package in Python 3.13. Edges in the network represented pairwise Pearson correlations that reached statistical significance (*p* < 0.05). The absolute value of the correlation coefficient was used as the edge weight, with no additional thresholding applied beyond statistical significance. Both positive and negative correlations were included and visually differentiated in the graphical output. The network was constructed as an undirected weighted graph using the spring-layout algorithm, allowing visualization of the strength and direction of associations among variables. Sex-stratified correlations were computed descriptively to explore potential sex-specific patterns; however, no formal statistical comparison between correlation coefficients (e.g., Fisher’s Z test) was performed. This network was conceived purely as a descriptive visualization of statistically significant correlations and was not intended to represent a psychometric or inferential network structure. Therefore, graph-theoretical indices such as centrality, density, or modularity were not computed, and no resampling-based validation was conducted. The figure serves as a visual complement to the correlation matrix, rather than as a structural model.

Differences in orthorexia-related scores across categorical variables were analyzed using one-way analysis of variance (ANOVA). When ANOVA yielded statistically significant results, post hoc comparisons were performed using Tukey’s HSD test. Effect sizes were estimated using partial eta squared (η^2^p), interpreted as small (≥0.01), moderate (≥0.06), or large (≥0.14). For variables with two categories, Cohen’s d was calculated.

To identify distinct orthorexia profiles, a hierarchical cluster analysis followed by a non-hierarchical K-means procedure was conducted. For this analysis, we used exclusively the standardized z-scores of the three main orthorexia-related dimensions: HeOr, OrNe, and the global EHQ score. The EHQ subscales were not included in the clustering variables to avoid multicollinearity and conceptual redundancy. These subscales were used only in subsequent within-cluster partial correlation analyses. Initially, Ward’s agglomeration method with squared Euclidean distance was applied to explore the structure of the data and determine the optimal number of clusters. This hierarchical step was used to examine the underlying structure of the dataset and to obtain an empirical indication of the most appropriate number of clusters through inspection of the dendrogram and the agglomeration coefficients. The inspection of the dendrogram, together with the proportion of explained variance, supported the solution of three clusters. Once this number was identified, a non-hierarchical K-means procedure was applied to refine and optimize the final partition, as K-means allows iterative reallocation of cases to improve within-cluster homogeneity. Based on this information, a K-means clustering procedure was subsequently performed, fixing the number of clusters at three. The use of standardized scores allowed for direct comparison across variables with different measurement scales. The decision to retain three clusters was also supported by previous studies on orthorexia that reported similar three-profile structures using comparable measures and analytic approaches [22,26,27]. No external cluster validation indices (such as the silhouette coefficient or Calinski–Harabasz index) were calculated. Given the exploratory nature of this analysis, cluster selection was based on hierarchical structure, explained variance, and theoretical interpretability consistent with prior orthorexia research. No resampling-based or bootstrap procedures were conducted to assess the stability of the cluster solution.

Finally, partial correlations stratified by cluster were calculated between EHQ scores (total and subscales), OrNe, and HeOr, adjusting for age, sex, and BMI. This procedure was used to explore the internal consistency of the EHQ within each profile and to examine the differential relationships between healthy and pathological dimensions of orthorexia. These within-cluster partial correlations were not intended to replicate or validate the cluster structure, but rather to examine the internal relationships among the EHQ subscales and their links with OrNe and HeOr once individuals were grouped according to their overall orthorexia profile. Because the clusters were derived only from the three global orthorexia indicators (EHQ total, OrNe, HeOr), and not from the EHQ subscales, stratified correlations allow a meaningful exploration of how these subcomponents be-have within each profile, providing complementary construct-level insight.

All analyses were performed using IBM SPSS Statistics version 25 (IBM Corp., Armonk, NY, USA). Statistical significance was set at *p* < 0.05. No a priori power analysis was conducted. The sample size was determined pragmatically based on the total number of eligible participants available during the recruitment period.

## 3. Results

Table 1 shows the descriptive characteristics of the sample by sex. The sample consisted of 190 participants (101 women and 89 men), with a mean age of 23.16 years (SD = 5.13) and a mean BMI of 23.69 (SD = 2.73). Statistically significant differences were observed between sexes in body mass index (*p* = 0.030), with higher values in men. Likewise, significant differences were found in the type of sport practiced (*p* = 0.013), participation in official competitions (*p* = 0.013), and the type of supplement consumed (*p* = 0.025). No significant differences were found between sexes in age, frequency or duration of training, overall supplement consumption, or scores on the EHQ and TOS questionnaires.

**Figure 1:**
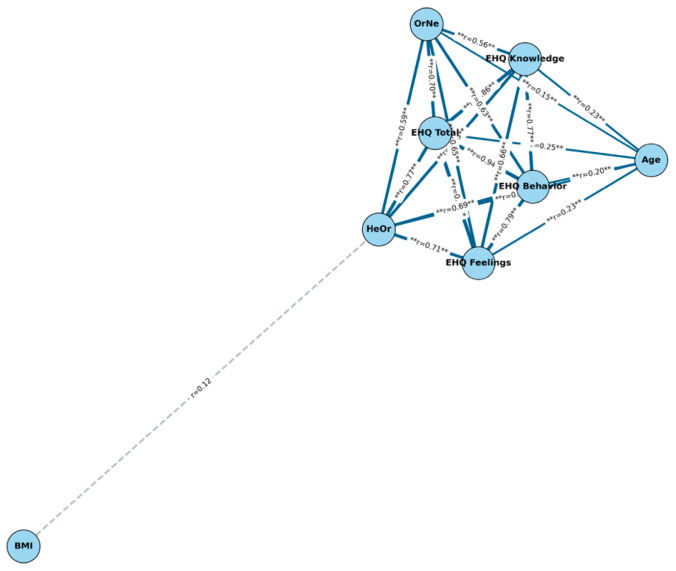
Semantic network of correlations. Each node represents a variable, and the links (edges) represents statistically significant correlations between pairs of variables. The thickness of the edges reflects the strength of the correlation, while the line style indicates the level of statistical significance (solid lines for *p* < 0.01; dashed lines for *p* < 0.05; gray lines for non-significant correlations). Asterisks indicate statistically significant differences.

The correlation coefficients (r) between age, participants’ BMI, and orthorexia questionnaire scores for the total sample and by sex are presented in Table 2 and Figure 1. Age was positively correlated with the total EHQ score (r = 0.251, *p* < 0.01), as well as with its subscales, both in the overall sample and in women. Likewise, positive correlations were observed between age and the OrNe (r = 0.149, *p* < 0.05) and HeOr (r = 0.296, *p* < 0.01) scores, the latter being particularly high in men (r = 0.365, *p* < 0.01). Regarding BMI, positive correlations were only identified in men with the total EHQ score (r = 0.221, *p* < 0.05), knowledge (r = 0.214, *p* < 0.05), and OrNe (r = 0.222, *p* < 0.05). On the other hand, the scores on the different questionnaires showed high correlations with each other: the total EHQ score was strongly associated with OrNe (r = 0.700, *p* < 0.01) and HeOr (r = 0.768, *p* < 0.01), and these two dimensions were also correlated with each other (r = 0.588, *p* < 0.01).

The mean differences in EHQ, OrNe, and HeOr scores according to diet, characteristics of the sport practiced, and supplement use, by sex, are included in Supplementary Table S1 and presented in Table 3. The ANOVA analysis showed significant differences in orthorexia-related scores across several sport- and diet-related variables. To aid interpretation, effect sizes were considered alongside statistical significance. Across variables, η^2^p and Cohen’s d values generally indicated small to moderate practical differences, with a few large effects—particularly in associations related to sport type, supplement use, and participation in competitions. These effect sizes provide additional context, suggesting that while some group differences are substantial, others represent more modest variations in orthorexia-related tendencies. Consistent with conventional benchmarks, many of the observed effects fell within the small-effect range, and their interpretation should be understood accordingly. Regarding diet type, significant differences were found in the total EHQ score (*p* = 0.048, η^2^p = 0.042), especially in the knowledge subscale (*p* = 0.004, η^2^p = 0.070). These effects were more evident in women, with moderate effect sizes for the total score (η^2^p = 0.072) and the knowledge subscale (η^2^p = 0.110, approaching the large-effect threshold). No significant differences were observed in men. The type of sport practiced was significantly associated with the EHQ total score (*p* = 0.003, η^2^p = 0.093), knowledge (*p* = 0.003, η^2^p = 0.092), behaviors (*p* = 0.015, η^2^p = 0.073), and feelings (*p* = 0.026, η^2^p = 0.066), with small to moderate effect sizes. The largest effects were observed in women, especially for HeOr (*p* = 0.002, η^2^p = 0.176). Regarding the frequency of weekly training sessions, only a weak association with HeOr was found in the total sample (*p* = 0.049, η^2^p = 0.032), and no significant effects were detected when stratified by sex. Training duration showed no significant association with any of the orthorexia variables, with trivial effect sizes across all groups. The type of supplement consumed was significantly associated with the EHQ total score (*p* = 0.037, η^2^p = 0.212) and the knowledge subscale (*p* = 0.008, η^2^p = 0.282), showing medium to large effect sizes in men, particularly for knowledge (η^2^p = 0.400). Regarding participation in official competitions, significant differences were observed in the total EHQ (*p* = 0.003, d = –0.447), knowledge (*p* = 0.016, d = –0.348), behaviors (*p* = 0.007, d = –0.409), feelings (*p* = 0.006, d = –0.416), and HeOr (*p* < 0.001, d = –0.585). Among women, all associations were significant (*p* < 0.01), with large effect sizes, especially in HeOr (d = –0.842), suggesting that non-competing women scored significantly higher on healthy orthorexia.

Post hoc analysis showed that participants following a vegetarian diet scored significantly lower on the Eating Habits Questionnaire (EHQ) compared to those following a Mediterranean diet (*p* = 0.035). However, on the knowledge subscale, vegetarians showed significantly higher scores (*p* = 0.002). Regarding the sport practiced, participants in fitness obtained significantly higher total EHQ scores than those who played handball (*p* = 0.034) or soccer (*p* = 0.048). Weekly training frequency was also associated with higher HeOr scores. Participants who trained five or more times per week reported a stronger non-pathological interest in healthy eating compared to those training three to four times per week (*p* = 0.037). This reflects an adaptive pattern consistent with the conceptualization of HeOr as a healthy engagement with nutrition rather than a pathological tendency. Finally, the type of supplement consumed showed significant differences only on the knowledge subscale of the EHQ. Participants who consumed vitamins obtained significantly higher scores on the knowledge subscale of the EHQ compared to those who consumed protein (*p* = 0.035) or other supplements (*p* = 0.035).

**Figure 2:**
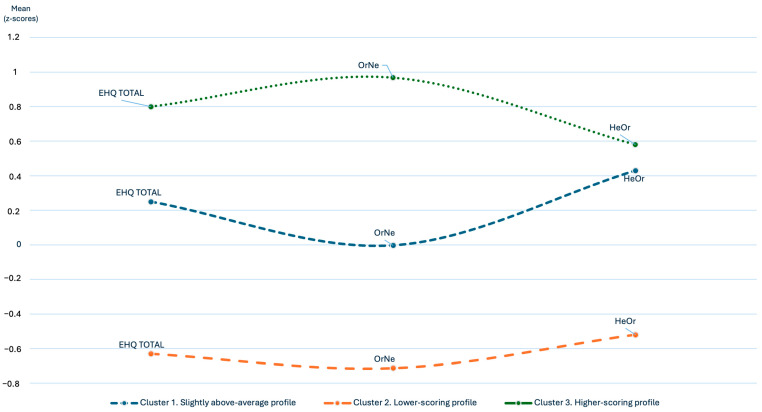
Orthorexic profiles identified though cluster analysis. Standardized (Z) distribution of scores in healthy orthorexia (HeOr), orthorexia nervosa (OrNe), and the Eating Habits Questionnaire (EHQ) across the three identified profiles: Cluster 1. Slightly above-average profile; Cluster 2. Lower-scoring profile; and Cluster 3. Higher-scoring profile.

Cluster analysis identified three distinct participant profiles based on their standardized scores on the HeOr, OrNe, and EHQ (Figure 2). The Slightly above-average profile (n = 83) was characterized by HeOr and EHQ scores that were marginally above the mean (Z = +0.43 and Z = +0.20, respectively), while OrNe scores remained very close to the sample mean (Z ≈ 0). The Lower-scoring profile (n = 75) showed scores below the mean on all three variables (Z = −0.95 on the HeOr, Z = −0.71 in OrNe, and Z = −0.88 in EHQ), while the Higher-scoring profile (n = 32) presented high scores in all of them (Z = +1.10, +1.68, and +1.43, respectively).

**Figure 3:**
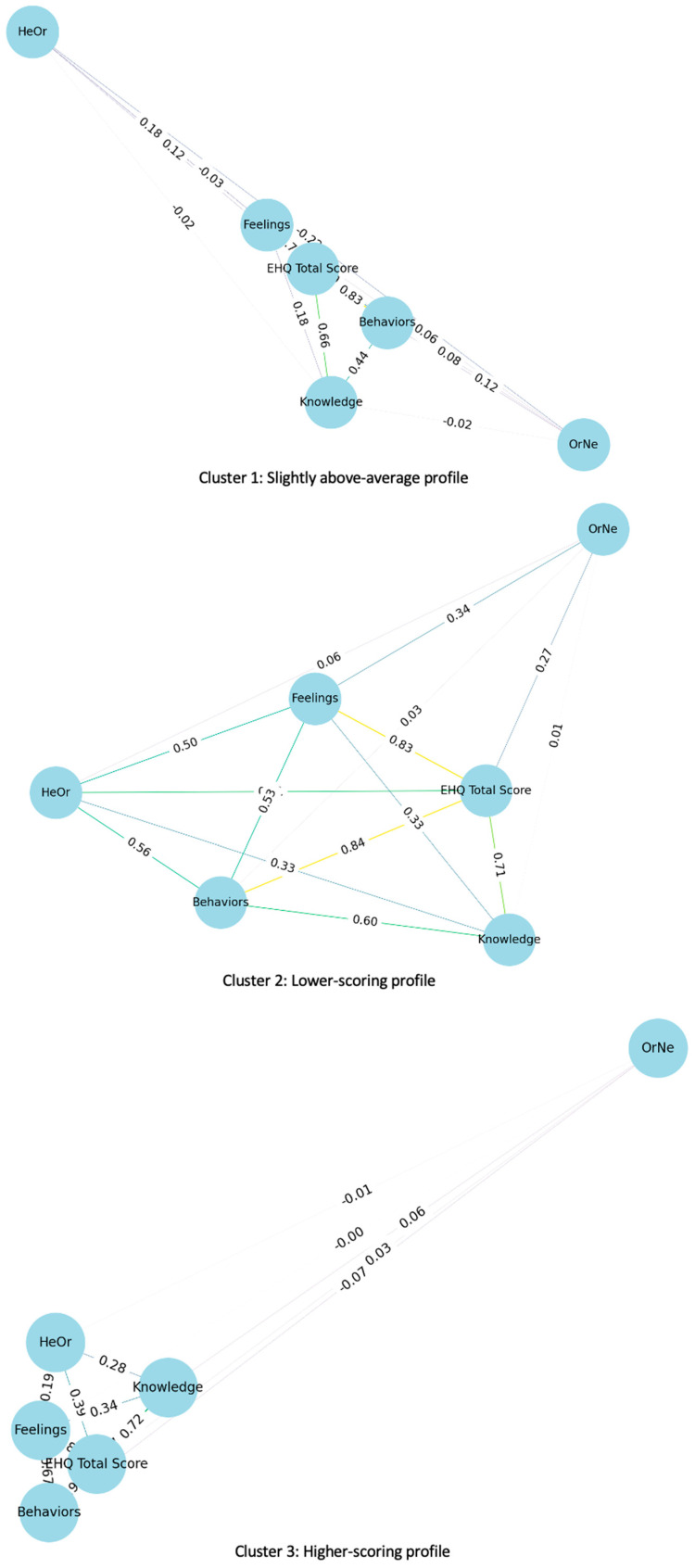
Partial correlation networks of orthorexia scores by cluster profiles. Visualization of partial correlations adjusted for age, sex, and BMI among scores from the Eating Habits Questionnaire (EHQ: total, knowledge, behaviors, and feelings), orthorexia nervosa (OrNe), and healthy orthorexia (HeOr), stratified by cluster. From left to right: Cluster 1 (Slightly above-average profile), Cluster 2 (Lower-scoring profile), and Cluster 3 (Higher-scoring profile). The thickness and color of the lines represent the strength and direction of correlations (positive in green/blue, negative in red/gray). Only statistically significant correlations (*p* < 0.05) are displayed.

The descriptive characteristics of the clusters are included in Supplementary Table S2. Differences between clusters were statistically significant for all questionnaire scores (*p* < 0.001; ES between 0.503 and 0.684). Significant differences were also observed in age (*p* < 0.001) and in variables such as the type of sport practiced (*p* < 0.001), training frequency (*p* = 0.013), participation in official competitions (*p* < 0.001), and supplement use (*p* = 0.004), with moderate effect sizes. No significant differences were found between clusters regarding sex or type of diet followed. On the other hand, partial correlations between clusters, adjusted for age, sex, and BMI (Figure 3, Supplementary Table S3), showed high internal consistency of the EHQ across all three profiles, especially between the total score and the behaviors subscale (r = 0.832 to 0.896; *p* < 0.01). The associations between OrNe and EHQ were low or nonsignificant in the Slightly above-average and Higher-scoring profiles, while in the Lower-scoring profile, significant correlations were observed with the feelings subscale (r = 0.335; *p* < 0.01) and with the total EHQ (r = 0.273; *p* < 0.05). The HeOr showed moderate correlations with EHQ scores in the Lower-scoring profile (r = 0.333–0.610; *p* < 0.01) and weaker relationships in the Higher-scoring profile, with only its association with the behavioral subscale being significant (r = 0.453; *p* < 0.05). In the Slightly above-average profile, the only significant correlation was negative, between HeOr and OrNe (r = −0.223; *p* < 0.05).

## 4. Discussion

The present study analyzed the associations between orthorexia in its healthy (HeOr) and pathological (OrNe) dimensions and sociodemographic, sports, and dietary variables in a sample of physically active young adults. The findings confirm that orthorexia is a heterogeneous and multifactorial phenomenon, in which both individual and contextual factors appear to contribute to its expression.

First, the association between age and orthorexia-related scores suggests that older participants tended to report more structured or rigid approaches to healthy eating. This result is consistent with the review by McInerney et al., who similarly reported higher levels of orthorexic behaviors in adults compared to adolescents [28]. However, other longitudinal studies have described more variable trajectories, with a decrease in orthorexic behaviors at older ages, possibly due to a lower level of social pressure or a readjustment of life priorities [29]. In this sense, our findings may also reflect characteristics typical of physically active young adults, a life stage in which concerns about health and self-care tends to be more pronounced.

The finding that BMI is only associated with orthorexia only in men should be interpreted with caution. Most male participants in our sample practiced fitness or soccer—sports with a strong strength- and muscle-oriented focus—which may not reflect the characteristics of men participating in other athletic disciplines. Therefore, this association may not be generalizable to all male athletes. Previous studies have shown that concern about “pure” eating may be modulated by body perception, with men being more sensitive to the relationship between weight, muscle mass, and athletic performance [30]. The absence of this association in women is consistent with that described by Lucka et al., who note that the determinants of orthorexia in women seem to be more linked to ideals of health and thinness than to body weight [31]. This reinforces the hypothesis of a gender dimension in the expression of orthorexia highlighting the need for tailored prevention strategies.

Regarding dietary habits, the fact that vegetarian participants displayed higher nutritional knowledge, but lower orthorexia scores suggests that nutritional literacy can coexist with healthy and flexible eating patterns. This result supports the idea that greater nutritional literacy does not necessarily translate into dysfunctional behaviors but can coexist with conscious and balanced dietary choices. These findings are consistent with the systematic review by Brytek-Matera, which highlights that vegetarianism, when motivated by ethical or environmental reasons rather than strict dietary control, does not increase orthorexia risk [32]. However, other studies have found a higher prevalence of orthorexia in strict vegetarians, suggesting that the underlying motivation and sociocultural context could be key factors in its onset [33].

Participants involved in fitness presented the highest orthorexia scores, a pattern consistent with prior research linking this population to greater risk driven by aesthetic pressure and performance-oriented demands [34,35,36]. Team sports such as soccer and handball showed comparatively lower scores, reinforcing the idea that the individualized and aesthetic sports environment increases vulnerability [37]. Furthermore, nutritional supplement use—especially among men—has been associated with a higher level of nutritional concern and greater rigidity in eating patterns. Previous studies have shown that supplement use and orthorexia-related behaviors frequently may reflect, particularly in performance-oriented sport environments [30,38,39]. These associations suggest that supplement use co-occur with broader patterns of nutrition-related preoccupation, although the cross-sectional design does not allow conclusions about directionality. In this context, supplement use may function as an indicator of heightened nutritional concern in certain athletic populations.

Taken together, these findings support the notion that orthorexia emerges from the interaction between individual factors (age, sex, BMI), dietary choices, and the sports context, and highlighting the need for a multifactorial approach to its detection and prevention. It should also be considered that cultural health ideals in Spain, particularly those related to the Mediterranean dietary pattern, may influence how individuals perceive and report healthy eating behaviors, potentially shaping both adaptive practices and more rigid tendencies captured by EHQ and TOS scores.

The results of the cluster analysis allow us to identify three distinct profiles based on standardized scores in the HeOr, OrNe, and EHQ dimensions, reinforcing the idea that ON should not be understood as a homogeneous phenomenon, but rather as a multidimensional construct with distinct patterns that coexist across individuals. The high-orthorexic profile, characterized by high scores in all three dimensions, reflects a configuration in which rigid behaviors and high emotional involvement accompany the obsessive concern with healthy eating. This pattern is consistent with previous studies that point to the coexistence of dysfunctional cognitions and restrictive behaviors as the clinical core of orthorexia nervosa [5,40].

The negative correlation between HeOr and OrNe in this group suggests a possible tension between healthy interest and the emergence of obsessive thoughts, although this interpretation should be viewed cautiously within a cross-sectional framework. In contrast, the lower-scoring profile showed low scores in all dimensions, but with significant correlations between OrNe and the feelings subscale of the EHQ, indicating that even in the absence of rigid behaviors, emotional components linked to eating may persist. This finding reinforces the need to consider the affective dimension in the assessment of orthorexia, as recent reviews have pointed out [41].

Furthermore, significant differences across clusters in variables such as the type of sport practiced, training frequency, and supplement use indicate the influence of the sports context in relation to the presence of different orthorexia profiles. Individualized and aesthetic sports were more frequently observed in participants with elevated orthorexia profiles, whereas collective disciplines tended to show lower orthorexia scores. These patterns reflect associations rather than causal relationships and may relate to contextual factors within different sport environments [7]. The high internal consistency of the EHQ across all profiles, especially in the behavioral subscale, supports its usefulness as an assessment tool. However, the weak correlations between OrNe and EHQ in the most symptomatic profiles suggest that cognitive obsession may operate independently of observable behaviors, posing new challenges for early detection and clinical intervention.

An innovative aspect of this work is the observation of differential patterns of internal coherence between the EHQ, OrNe, and HeOr dimensions according to the identified profile. While in the higher-scoring cluster, a high level of consistency in behaviors is maintained, the dissociation of OrNe suggests that different profiles may display distinct configurations of cognitive and behavioral components. This separation between “behavior” and “cognition” may reflect a configuration in which cognitive rigidity regarding eating coexists with comparatively more flexible behaviors, a pattern that has been observed in some orthorexia-related profiles. A plausible hypothesis is that these profiles represent different configurations of orthorexia-related characteristics, without implying temporal progression.

From an applied perspective, identifying these profiles enables consideration of differentiated interventions. The slightly above-average profile, for example, could benefit from preventive strategies based on critical nutritional literacy and cognitive flexibility training. In contrast, the higher-scoring profile would require more structured clinical interventions, similar to those used for eating disorders. In a novel way, we propose that the resilient or lower-scoring profile could serve as a natural control group for exploring protective factors such as self-efficacy, intrinsic health motivation, and reduced exposure to toxic digital content related to the “perfect diet.”

Another contribution of this work is the descriptive correlation network included to visually summarize the relationships among orthorexia-related variables within each profile. Although the network does not allow us to draw conclusions about centrality, structural properties, or dynamic behavior, it provides a useful exploratory visualization of how questionnaire dimensions relate to each other. This descriptive approach may encourage future studies to apply more advanced psychometric or dynamic network models to orthorexia, which could help identify potential targets for intervention once validated through appropriate analytical procedures. Our results are consistent with studies highlighting the high prevalence of orthorexia in sports populations and healthcare students [28], but they provide a novel perspective by showing that the profiles differ not only in intensity but also in the internal structure of their correlations. This idea is consistent with recent work in psychopathology that suggests that disorders are not unitary entities, but rather dynamic symptomatic networks that vary between individuals [29].

These results have relevant applications in the prevention and detection of orthorexia. The identification of distinct profiles enables the design of targeted interventions based on the type of sport, supplement use, or type or intensity of sport involvement. Furthermore, the findings reinforce the usefulness of using multiple instruments simultaneously to discriminate between healthy and pathological orthorexia, a key aspect in avoiding the pathologizing of interest in a balanced diet [39].

Beyond the theoretical implications, our results also have clinical relevance. Athletes with elevated orthorexia scores—particularly those involved in fitness or individual disciplines—may benefit from early screening focused on cognitive rigidity, emotional dis-comfort around food, and overcontrolled eating patterns. Integrating brief assessment tools, nutritional psychoeducation, stress-management strategies, and cognitive-flexibility training into sport settings may help reduce risk. Considering emerging evidence on the psychophysiological correlates of orthorexic tendencies, combining behavioral and psychological indicators could provide more comprehensive prevention approaches aimed at safeguarding athlete wellbeing [42].

### Limitations and Future Research

This study presents several limitations that should be considered when interpreting the findings. First, the cross-sectional design precludes any causal inference, and the use of a non-probability convenience sample recruited through gyms, sports clubs, and social media may introduce selection bias. Consequently, the sample may not fully represent the broader population of physically active young adults, and generalizability is limited. The exclusion of individuals with eating disorders or psychiatric diagnoses relied on self-report, which may reduce the accuracy of participant screening. In addition, weight, height, and dietary variables were self-reported, introducing potential recall and social desirability biases.

No a priori power analysis was conducted, and the final sample size was determined by feasibility rather than statistical optimization. Although comparable to previous orthorexia studies, the absence of a formal sample size justification limits the evaluation of statistical power. Participants were not evaluated separately during competitive and non-competitive periods, and because dietary patterns may become more restrictive around competitions, this factor cannot be ruled out as a source of variability.

Regarding statistical analyses, the large number of tests conducted without correction increases the risk of Type I error; therefore, small effects and *p*-values close to the significance threshold should be interpreted cautiously. Sex-stratified analyses were exploratory, and formal interaction tests (e.g., factorial ANOVAs or Fisher’s Z comparisons) were not performed due to small subgroup sizes. As such, potential sex differences should be interpreted with caution and examined more rigorously in future research.

Several limitations also apply to the clustering procedures. As with all exploratory clustering approaches, the choice of the three-cluster solution depends partly on interpretability. Replication is therefore needed to confirm the stability of the identified profiles. No internal validation indices or bootstrap-based stability analyses were conducted, restricting the assessment of cluster robustness. Furthermore, the semantic correlation network was purely descriptive and did not involve psychometric network modeling, centrality metrics, or statistical validation; therefore, it should not be interpreted as reflecting causal or structural relationships.

Future studies should incorporate longitudinal designs, probabilistic sampling strategies, formal power analyses, and validated diagnostic criteria for orthorexia. Additionally, the use of external cluster validation methods, stability analyses, and psychometric network models would strengthen the interpretability and replicability of orthorexia profiles. Exploring contextual factors—such as competition cycles, social media exposure, and broader sociocultural influences—would also deepen the understanding of orthorexia in physically active populations.

## 5. Conclusions

Cluster analysis confirms the heterogeneity of orthorexia and indicates that the identified profiles reflect different patterns that coexist in physically active adults. However, given the cross-sectional design, no temporal progression or causal transitions between profiles can be inferred. The associations observed with individual factors (e.g., age, BMI) and contextual variables (e.g., sport type, supplement use, or sport-related pressures) highlight the importance of studying orthorexia within specific sport environments.

Future research should incorporate longitudinal designs to examine the stability or variability of orthorexia-related patterns over time, as well as comparative analyses across diverse sport disciplines—including aesthetic, endurance, and strength-based sports—to better understand sport-specific vulnerabilities and protective factors. These approaches may contribute to more precise prevention and intervention strategies for athletes and physically active individuals in different sporting contexts.

## Figures and Tables

**Table 1:** Descriptive characteristics of the sample.

	Total		Females		Males		
	N	% or M (SD)	N	% or M (SD)	N	% or M (SD)	*p*
**Age**	190	23.16 (5.13)	101	23.29 (5.13)	89	23.01 (5.14)	0.681
**BMI**	190	23.69 (2.73)	101	23.30 (3.07)	89	24.14 (2.21)	**0.030**
Underweight	5	2.6	5	5.0	0	0	
Normal weight	123	64.7	63	62.4	60	67.4	
Overweight	60	31.6	31	30.6	29	32.6	
Obesity	2	1.1	2	2.0	0	0	
**Diet type**							0.136
Mediterranean	172	90.5	94	93.1	78	87.6	
Vegetarian	8	4.2	5	5.0	3	3.4	
Hyperproteic	7	3.7	2	2.0	5	5.6	
Others/no specific	3	1.6	0	0	3	3.4	
**Type of sport**							**0.013**
Running	9	4.7	2	2.0	7	7.9	
Handball	81	42.6	53	52.5	28	31.5	
Cycling	3	1.6	1	1.0	2	2.2	
Fitness	48	25.3	21	20.8	27	30.3	
Soccer	21	11.1	7	6.9	14	15.7	
Others	28	14.7	17	16.8	11	12.4	
**Frequency of weekly training sessions**							0.209
1 or 2	22	11.6	9	8.9	13	14.6	
3 or 4	113	59.5	58	57.4	55	61.8	
≥5	55	28.9	34	33.7	21	23.6	
**Training duration**							0.714
1–2 h	129	67.9	68	67.3	61	68.5	
2–3 h	31	16.3	19	18.8	12	13.5	
30–60 min	26	13.7	12	11.9	14	15.7	
>3 h	4	2.1	2	2.0	2	2.2	
**Participation in official competitions**							**0.013**
Yes	116	61.1	70	69.3	46	51.7	
No	74	38.9	31	30.7	43	48.3	
**Consumption of supplements**							0.245
Yes	40	21.1	18	17.8	22	24.7	
No	150	78.9	83	82.2	67	75.3	
**Type of supplement**	39						**0.025**
Proteins	22	11.6	6	5.9	16	18.0	
Vitamins	8	4.2	6	5.9	2	2.2	
Proteins y vitamins	2	1.1	0	0	2	2.2	
Others	7	3.7	5	5.0	2	2.2	
**EHQ**							
Total	190	40.4 (10.59)	101	40.40 (11.77)	89	40.40 (9.12)	0.996
Behavior	190	14.71 (4.26)	101	14.75 (4.77)	89	14.66 (3.62)	
Knowledge	190	8.65 (2.81)	101	8.47 (3.03)	89	8.87 (2.54)	
Feelings	190	12.48 (3.42)	101	12.66 (3.74)	89	12.28 (3.02)	
**TOS**							
OrNe	190	4.00 (4.05)	101	4.48 (4.47)	89	3.46 (3.46)	0.214
HeOr	190	11.45 (6.05)	101	11.49 (6.20)	89	11.40 (5.90)	0.927

BMI: Body Mass Index; EHQ: Eating Habits Questionnaire; TOS: Teruel Orthorexia Scale; OrNe: Orthorexia nervosa; HeOr: Healthy Orthorexia. Data are shown as frequencies and percentages, except for age, BMI, and EHQ and TOS questionnaire scores, which are shown as Mean (M) ± Standard Deviation (SD). *p*-value indicates differences between sexes. In bold: statistically significant relationships.

**Table 2:** Correlation coefficients (r) between age and BMI of participants and orthorexia questionnaire scores.

		Age	EHQ Total Score	Knowledge	Behaviors	Feelings	OrNe	HeOr
**EHQ Total Score**	Total	0.251 **						
	Female	0.236 *						
	Male	0.247 *						
**Knowledge**	Total	0.232 **	0.860 **					
	Female	0.213 *	0.869 **					
	Male	0.192	0.862 **					
**Behaviors**	Total	0.195 **	0.935 **	0.772 **				
	Female	0.189	0.961 **	0.815 **				
	Male	0.178	0.901 **	0.707 **				
**Feelings**	Total	0.233 **	0.886 **	0.659 **	0.785 **			
	Female	0.254 *	0.914 **	0.661 **	0.823 **			
	Male	0.221 *	0.900 **	0.673 **	0.716 **			
**OrNe**	Total	0.149 *	0.700 **	0.557 **	0.634 **	0.652 **		
	Female	0.105	0.766 **	0.623 **	0.720 **	0.697 **		
	Male	0.190	0.626 **	0.482 **	0.519 **	0.600 **		
**HeOr**	Total	0.296 **	0.768 **	0.631 **	0.690 **	0.715 **	**0.588** **	
	Female	0.240 *	0.792 **	0.640 **	0.772 **	0.721 **	0.639 **	
	Male	0.365 **	0.740 **	0.626 **	0.478 **	0.714 **	0.542 **	
**BMI**	Total	0.125	0.070	0.048	0.045	0.105	0.049	0.009
	Female	0.021	−0.003	−0.056	−0.024	0.065	−0.027	−0.061
	Male	0.253 *	0.221 *	0.214 *	0.194	0.213 *	0.222 *	0.127

** The correlation is significant at the 0.01 level (bilateral). * The correlation is significant at the 0.05 level (bilateral). In bold: statistically significant relationships.

**Table 3:** Analysis of variance (ANOVA) testing mean differences in EHQ, OrNe, and HeOr scores by diet, characteristics of the sport performed and use of supplements, by gender.

		EHQ Total Score		Knowledge		Behaviors		Feelings		OrNe		HeOr	
		*p*	ES	*p*	ES	*p*	ES	*p*	ES	*p*	ES	*p*	ES
**Diet type**	Total	**0.048**	0.042	**0.004**	0.070	0.120	0.031	0.092	0.034	0.561	0.011	0.069	0.037
	Female	**0.026**	0.072	0.003	0.110	0.138	0.040	0.091	0.048	0.155	0.037	**0.042**	0.063
	Male	0.641	0.019	0.462	0.030	0.584	0.022	0.325	0.040	0.333	0.039	0.520	0.026
**Type of sport**	Total	**0.003**	0.093	**0.003**	0.092	**0.015**	0.073	**0.026**	0.066	0.380	0.028	**<0.001**	0.159
	Female	**0.044**	0.111	0.075	0.098	0.095	0.093	**0.041**	0.113	0.619	0.036	**0.002**	0.176
	Male	0.121	0.098	0.093	0.106	0.211	0.081	0.554	0.046	0.190	0.084	**0.014**	0.155
**Frequency of weekly training sessions**	Total	0.091	0.025	0.101	0.023	0.101	0.024	0.112	0.023	0.481	0.008	**0.049**	0.032
	Female	0.240	0.029	0.538	0.013	0.361	0.021	0.130	0.041	0.205	0.032	0.107	0.045
	Male	0.209	0.036	**0.047**	0.069	0.179	0.039	0.671	0.009	0.051	0.067	0.219	0.035
**Training duration**	Total	0.975	0.001	0.396	0.016	0.848	0.004	0.931	0.002	0.671	0.008	0.519	0.012
	Female	0.807	0.010	0.760	0.012	0.820	0.009	0.498	0.024	0.691	0.015	0.639	0.017
	Male	0.494	0.028	0.054	0.086	0.303	0.042	0.899	0.007	0.357	0.037	0.596	0.022
**Type of supplement**	Total	**0.037**	0.212	**0.008**	0.282	0.183	0.128	0.099	0.161	0.753	0.033	0.624	0.048
	Female	0.253	0.178	0.133	0.251	0.447	0.109	0.116	0.265	0.964	0.005	0.645	0.061
	Male	0.121	0.270	**0.024**	0.400	0.234	0.206	0.332	0.169	0.583	0.100	0.761	0.061
**Participation in official competitions**	Total	**0.003**	−0.447	**0.016**	−0.348	**0.007**	−0.409	**0.006**	−0.416	0.175	−0.203	**<0.001**	−0.585
	Female	**<0.001**	−0.749	**0.007**	−0.597	**0.002**	−0.679	**<0.001**	−0.753	0.051	−0.425	**<0.001**	−0.842
	Male	0.494	−0.146	0.815	−0.049	0.503	−0.143	0.534	−0.133	0.753	−0.067	0.072	−0.383

EHQ: Eating Habits Questionnaire; OrNe: Orthorexia nervosa; HeOr: Healthy Orthorexia. Effect sizes (ES) are shown as η^2^p, except for Participation in official competitions, which is shown as Cohen’s d. In bold: statistically significant relationships.

## Data Availability

The data presented in this study are available on request from the corresponding author due to privacy reasons.

## Supplementary Materials

The following supporting information can be downloaded at: https://www.mdpi.com/article/10.3390/nu17243814/s1, Supplementary Table S1. Mean differences (SD) in EHQ, OrNe, and HeOr scores by diet, characteristics of the sport performed and use of supplements, by gender. Supplementary Table S2. Descriptive characteristics of the clusters. Supplementary Table S3. Cluster-specific partial correlation coefficients (r) among EHQ total and subscale scores, OrNe, and HeOr, adjusted for sex, age, and BMI.
