# Supplementary material for: Orthorexia Profiles in Athletes: A Multidimensional Analysis Using the Eating Habits Questionnaire (EHQ) and the Teruel Orthorexia Scale (TOS)
Source: Nutrients. 2025 Dec 5;17(24):3814. doi: 10.3390/nu17243814 (PMC12735428; doi:10.3390/nu17243814)
Supplement: Supplementary file 1 [file nutrients-17-03814-s001.zip › nutrients-3989902-supplementary/Supplementary Table S1. Mean differences (SD) in EHQ, OrNe, and HeOr scores by diet, characteristics of the sport performed and use of supplements, by gender..pdf]

Supplementary Table S1: Mean differences (SD) in EHQ, OrNe, and HeOr scores by diet, characteristics of the sport performed and use of supplements, by gender.

				**EHQ Total Score**				**Behaviors**			**Knowledges**			**Feelings**			**OrNe**			**HeOr**		
			**YES**		**NO**			**YES**	**NO**		**YES**	**NO**		**YES**	**NO**		**YES**	**NO**		**YES**	**NO**	
			**n**	**M(SD)**	**n**	**M(SD)**	**p**	**M(SD)**	**M(SD)**	**p**	**M(SD)**	**M(SD)**	**p**	**M(SD)**	**M(SD)**	**p**	**M(SD)**	**M(SD)**	**p**	**M(SD)**	**M(SD)**	**p**
**Diet type**	**Mediterranean**	Total	172	39.82 (10.48)	18	45.94 (10.30)	**0.019**	8.45 (2.72)	10.61 (2.99)	**0.002**	14.49 (4.26)	16.83 (3.73)	**0.026**	12.36 (3.42)	13.67 (3.31)	0.123	2.92 (4.01)	4.72 (4.51)	0.428	11.11 (6.04)	14.67 (5.19)	**0.017**
		Female	94	39.55 (11.41)	7	51.71 (11.43)	**0.008**	8.20 (2.84)	12.00 (3.42)	**0.001**	14.50 (4.68)	18.14 (4.98)	0.051	12.45 (3.70)	15.57 (3.21)	**0.032**	4.24 (4.37)	7.57 (5.00)	0.057	11.06 (6.10)	17.14 (4.88)	**0.012**
		Male	78	40.14 (9.28)	11	42.27 (8.00)	0.417	8.74 (2.55)	9.73 (2.45)	0.232	14.47 (3.71)	16.00 (2.61)	0.192	12.26 (3.06)	12.45 (2.88)	0.840	3.54 (3.51)	2.91 (3.21)	0.576	11.17 (6.01)	13.09 (4.95)	0.314
	**Vegetarian**	Total	8	50.13 (12.50)	182	39.97 (10.33)	**0.008**	12.00 ((3.82)	8.51 (2.68)	**<0.001**	17.88 (4.70)	14.57 (4.20)	**0.031**	15.25 (3.37)	12.36 (3.38)	**0.019**	5.63 (5.15)	3.93 (4.00)	0.247	16.50 (4.78)	11.23 (6.01)	**0.015**
		Female	5	53.00 (13.73)	96	39.74 (11.37)	**0.013**	12.60 (3.85)	8.25 (2.84)	**0.001**	18.60 (5.86)	14.55 (4.66)	0.064	16.00 (3.81)	12.49 (3.68)	**0.040**	7.20 (5.81)	4.33 (4.38)	0.163	17.00 (5.87)	11.20 (6.11)	**0.041**
		Male	3	45.33 (10.69)	86	40.23 (9.01)	0.344	11.00 (4.36)	8.79 (2.46)	0.140	16.67 (2.31)	14.59 (3.64)	0.332	14.00 (2.65)	12.22 (3.03)	0.318	3.00 (3.00)	3.48 (3.49)	0.816	15.67 (3.06)	11.26 (5.93)	0.205
	**Hyperproteic**	Total	7	41.70 (7.68)	183	40.35 (10.69)	0.739	9.43 (1.72)	8.62 (2.84)	0.458	15.86 (2.73)	14.67 (4.30)	0.469	11.71 (2.93)	12.51 (3.44)	0.545	3.29 (3.95)	4.03 (4.06)	0.636	12.86 (5.64)	11.39 (6.07)	0.531
		Female	2	48.50 (0.71)	99	40.23 (11.84)	0.328	10.50 (2.12)	8.42 (3.03)	0.339	17.00 (2.83)	14.71 (4.80)	0.504	14.50 (0.71)	12.63 (3.77)	0.486	8.50 (3.54)	4.39 (4.46)	0.200	17.50 (2.12)	11.36 (6.20)	0.167
		Male	5	39.00 (7.48)	84	40.49 (9.24)	0.726	9.00 (1.58)	8.86 (2.59)	0.452	15.40 (2.88)	14.62 (3.67)	0.642	10.60 (2.70)	12.38 (3.02)	0,202	1.20 (1.10)	3.60 (3.51)	**0.003**	11.00 (5.61)	11.43 (5.95)	0.876
	**Others/no specific**	Total	3	44.67 (6.66)	187	40.33 (10.63)	0.483	9.67 (1.53)	8.63 (2.82)	0.530	16.33 (3.21)	14.68 (4.27)	0.254	14.00 (2.00)	12.46 (3.43)	0.440	5.67 (4.62)	3.97 (4.05)	0.474	14.00 (5.29)	11.41 (6.06)	0.462
		Female	0																			
		Male	3	44.67 (6.66)	187	40.33 (10.63)	0.483	9.67 (1.53)	8.63 (2.82)	0.530	16.33 (3.21)	14.68 (4.27)	0.254	14.00 (2.00)	12.46 (3.43)	0.440	5.67 (4.62)	3.97 (4.05)	0.474	14.00 (5.29)	11.41 (6.06)	0.462
**Type of sport**	**Running**	Total	9	41.00 (6.40)	181	40.37 (10.76)	0.862	9.33 (2.45)	8.62 (2.83)	0.458	14.56 (3.61)	14.72 (4.309	0.911	12.33 (3.04)	12.49 (3.44)	0.893	2.56 (2.45)	4.07 (4.11)	0.274	13.33 (3.24)	11.35 (6.14)	0.119
		Female	2	37.00 (12.50)	99	40.46 (11.80)	0.682	7.50 (2.12)	8.48 (3.05)	0.651	15.00 (7.07)	14.75 (4.76)	0.941	11.00 (7.07)	12.70 (3.70)	0.528	2.50 (3.54)	4.52 (4.49)	0.531	12.50 (6.36)	11.46 (6.23)	0.816
		Male	7	42.14 (3.81)	82	40.26 (9.44)	0.306	9.86 (2.41)	8.78 (2.55)	0.285	14.43 (2.99)	14.68 (3.68)	0.859	12.71 (1.80)	12.24 (3.11)	0.695	2.57 (2.44)	3.54 (3.54)	0.965	13.57 (2.64)	11.22 (6.07)	0.073
	**Handball**	Total	81	38.06 (10.67)	109	42.14 (10.23)	**0.008**	7.99 (2.84)	9.15 (2.70)	**0.005**	13.88 (4.38)	15.33 (4.08)	**0.020**	11.96 (3.24)	12.87 (3.51)	0.070	3.79 (4.29)	4.16 (3.88)	0.540	9.74 (6.25)	12.72 (5.59)	**<0.001**
		Female	53	37.79 (11.56)	48	43.27 (11.41)	**0.019**	7.74 (2.96)	9.27 (2.91)	**0.010**	13.81 (4.82)	15.79 (4.54)	**0.037**	11.98 (3.46)	13.42 (3.93)	0.054	3.98 (4.57)	5.02 (4.33)	0.245	9.64 (6.41)	13.52 (5.32)	**0.001**
		Male	28	38.57 (8.83)	61	41.25 (9.20)	0.201	8.46 (2.56)	9.05 (2.53)	0.136	14.00 (3.46)	14.97 (3.67)	0.244	11.93 (2.84)	12.44 (3.11)	0.459	3.43 (3.76)	3.48 (3.36)	0.953	9.93 (6.04)	12.08 (5.76)	0.110
	**Cycling**	Total	3	50.33 (9.71)	187	40.24 (10.55)	0.102	12.00 (3.00)	8.60 (2.78)	**0.019**	17.33 (4.04)	14.67 (4.26)	0.283	14.33 (2.52)	12.45 (3.43)	0.346	8.00 (3.46)	3.94 (4.03)	0.085	17.00 (3.46)	11.36 (6.04)	0.109
		Female	1	48.00	100	40.32 (11.81)	0.519	12.00	8.43 (3.02)	0.242	15.00	14.75 (4.79)	0.959	14.00	12.65 (3.76)	0.722	6.00	4.46 (4.49)	0.734	19.00	11.41 (6.18)	0.225

		Male	2	51.50 (13.44)	87	40.15 (8.95)	0.082	12.00 (4.24)	8,79 (2.48)	0.078	18.50 (4.95)	14.57 (3.57)	0.130	14.50 (3.54)	12.23 (3.01)	0.296	9.00 (5.66)	3.33 (3.34)	**0.021**	16.00 (4.24)	11.30 (5.91)	0.268
	**Fitness**	Total	48	43.69 (9.76)	142	39.29 (10.66)	**0.012**	9.31 (2.38)	8.43 (2.91)	**0.039**	15.98 (4.17)	14.28 (4.21)	**0.017**	13.44 (3.50)	12.16 (3.34)	**0.025**	4.54 (3.78)	3.82 (4.14)	0.285	14.27 (4.89)	10.49 (6.11)	**<0.001**
		Female	21	46.10 (12.27)	80	38.90 (11.24)	**0.012**	9.57 (3.01)	8.18 (2.98)	0.059	17.00 (4.78)	14.16 (4.62)	**0.014**	14.52 (44.38)	12.18 (3.42)	**0.010**	5.95 (4.65)	4.09 (4.37)	0.089	15.38 (5.47)	10.46 (6.00)	**<0.001**
		Male	27	41.80 (6.92)	62	39.79 (9.92)	0.339	9.11 (1.78)	8.76 (2.81)	0.550	15.19 (3.52)	14.44 (3.66)	0.372	12.59 (2.39)	12.15 (3.26)	0.472	3.44 (2.52)	3.47 (3.82)	0.977	13.41 (4.29)	10.53 (6.31)	**0.034**
	**Soccer**	Total	21	35.95 (10.17	129	40.95 (10.54)	**0.041**	7.71 (2.85)	8.77 (2.79)	0.105	13.10 (3.51)	14.91 (4.31)	0.065	10.95 (3.54)	12.67 (3.37)	**0.029**	4.05 (4.18)	3.99 (4.05)	0.477	7.81 (5.71)	11.90 (5.95)	**0.002**
		Female	7	34.71 (10.28)	94	40.82 (11.82)	0.187	7.86 (2.97)	8.51 (3.04)	0.584	12.43 (3.95)	14.93 (4.80)	0.183	10.29 (3.30)	12.84 (3.73)	0.090	3.86 (3.76)	4.52 (4.53)	0.706	8.29 (4.61)	11.72 (6.26)	0.158
		Male	14	36.57 (10.45)	75	41.12 (8.74)	0.087	7.64 (2.90)	9.09 (2.42)	**0.049**	13.43 (3.37)	14.89 (3.64)	0.166	11.26 (3.73)	12.47 (2.85)	0.181	4.14 (4.50)	3.33 (3.26)	0.425	7.57 (6.33)	12.12 (5.57)	**0.007**
	**Others**	Total	28	43.61 (10.63)	162	39.85 (10.51)	0.083	9.57 (2.83)	8.49 (2.78)	0.061	15.93 (4.05)	14.50 (4.27)	0.101	13.36 (3.38)	12.33 (3.41)	0.072	3.68 (4.01)	4.06 (4.07)	0.651	13.07 (5.47)	11.17 (6.11)	0.124
		Female	17	43.76 (9.63)	84	39.71 (12.10)	0.197	9.53 (2.85)	8.25 (3.03)	0.112	15.82 (4.00)	14.54 (4.90)	0.312	13.59 (2.74)	12.48 (3.90)	0.084	4.59 (4.39)	4.45 (4.51)	0.910	13.18 (4.07)	11.14 (6.51)	0.103
		Male	11	43.36 (12.51)	78	39.99 (8.56)	0.404	9.64 (2.94)	8.76 (2.48)	0.285	16.09 (4.32)	14.46 (3.49)	0.163	13.00 (4.31)	12.18 (2.81)	0.552	2.27 (3.00)	3.63 (3.51)	0.226	12.91 (7.37)	11.19 (5.69)	0.369
**Frequency of weekly training sessions**	**1 or 2**	Total	22	40.05 (10.28)	168	40.45 (10.66)	0.868	8.73 (3.10)	8.64 (2.77)	0.895	14.23 (3.56)	14.77 (4.35)	0.573	12.32 (3.39)	12.51 (3.43)	0.809	4.32 (4.50)	3.96 (4.00)	0.969	11.55 (5.41)	11.43 (6.14)	0.936
		Female	9	37.27 (12.22)	92	40.71 (11.75)	0.399	7.89 (3.26)	8.52 (3.01)	0.552	13.56 (4.85)	14.87 (4.77)	0.433	11.78 (3.80)	12.75 (3.75)	0.460	2.44 (2.96)	4.67 (4.55)	0.154	9.67 (3.71)	11.66 (6.389	0.178
		Male	13	42.00 (8.68)	76	40.13 (9.22)	0.498	9.31 (2.98)	8.79 (2.47)	0.500	14.69 (2.43)	14.66 (3.80)	0.975	12.69 (3.17)	12.21 (3.01)	0.598	5.62 (5.01)	3.09 (3.02)	0.101	12.85 (6.14)	11.16 (5.87)	0.343
	**3 or 4**	Total	113	39.20 (10.78)	77	42.16 (10.10)	0.059	8.33 (2.79)	9.13 (2.79)	0.053	14.30 (4.39)	15.31 (4.01)	0.108	12.12 (3.34)	13.01 (3.49)	0.078	3.71 (3.89)	4.43 (4.28)	0.230	10.64 (6.08)	12.64 (5.82)	**0.025**
		Female	58	39.33 (11.87)	43	41.84 (11.62)	0.292	8.29 (3.11)	8.70 (2.93)	0.509	14.41 (4.91)	15.21 (4.60)	0.410	12.19 (3.60)	13.30 (3.88)	0.140	4.29 (4.48)	4.72 (4.50)	0.637	10.72 (6.09)	12.51 (6.27)	0.153
		Male	55	39.07 (9.61)	34	42.56 (7.94)	0.080	8.36 (2.44)	9.68 (2.53)	**0.017**	14.18 (3.81)	15.44 (3.19)	0.111	12.05 (3.07)	12.65 (2.94)	0.371	3.09 (3.05)	4.06 (4.03)	0.202	10.55 (6.14)	12.79 (5.29)	0.081
	**≥ 5**	Total	55	43.00 (10.00)	135	39.34 (10.67)	**0.030**	9.29 (2.66)	8.39 (2.83)	**0.045**	15.75 (4.13)	14.29 (4.25)	**0.032**	13.29 (3.52)	12.16 (3.34)	**0.038**	4.47 (4.24)	3.81 (3.97)	0.306	13.07 (5.97)	10.79 (5.97)	**0.018**
		Female	34	43.06 (11.33)	67	39.04 (11.85)	0.106	8.91 (2.85)	8.24 (3.10)	0.293	15.65 (4.50)	14.30 (4.87)	0.181	13.71 (3.85)	12.13 (3.60)	**0.046**	5.32 (4.68)	4.04 (4.33)	0.175	13.26 (6.63)	10.58 (5.81)	**0.039**
		Male	21	42.90 (7.64)	68	39.63 (9.45)	0.152	9.90 (2.26)	8.54 (2.55)	**0.031**	15.90 (3.56)	14.28 (3.57)	0.072	12.62 (2.87)	12.18 (3.08)	0.560	3.10 (3.03)	3.57 (3.60)	0.583	12.76 (4.86)	10.99 (6.16)	0.230
**Training duration**	**30-60 minutes**	Total	26	40.65 (9.93)	164	40.36 (10.72)	0.896	8.35 (2.48)	8.70 (2.86)	0.551	14.58 (4.03)	14.73 (4.30)	0.864	12.88 (3.53)	12.42 (3.41)	0.522	3.38 (3.31)	4.10 (4.16)	0.406	12.73 (6.19)	11.24 (6.02)	0.245
		Female	12	43.17 (11.73)	89	40.02 (11.80)	0.388	8.25 (3.02)	8.49 (3.04)	0.794	15.92 (4.76)	14.60 (4.78)	0.370	14.17 (4.02)	12.46 (3.68)	0.139	4.75 (3.91)	4.44 (4.56)	0.822	13.08 (5.93)	11.27 (6.24)	0.344
		Male	14	38.50 (7.90)	75	40.76 (9.34)	0.398	8.43 (2.03)	8.95 (2.63)	0.487	13.43 (3.01)	14.89 (3.69)	0.166	11.79 (2.72)	12.37 (3.08)	0.507	2.21 (2.23)	3.69 (3.61)	0.143	12.43 (6.62)	11.21 (5.79)	0.482

	**1-2 hours**	Total	129	40.17 (10.74)	61	40.89 (10.33)	0.665	8.55 (2.79)	8.87 (2.85)	0.467	14.72 (4.31)	14.69 (4.19)	0.961	12.41 (3.51)	12.64 (3.24)	0.668	4.23 (4.16)	3.51 (3.79)	0.251	11.01 (5.74)	12.38 (6.60)	0.073
		Female	68	40.18 (12.27)	33	40.85 (10.85)	0.789	8.53 (3.18)	8.33 (2.72)	0.762	14.66 (4.88)	14.94 (4.61)	0.785	12.49 (3.95)	13.03 (3.30)	0.495	4.75 (4.67)	3.91 (4.04)	0.378	11.09 (6.05)	12.30 (6.51)	0.358
		Male	61	40.16 (8.83)	28	40.93 (9.88)	0.716	8.57 (2.31)	9.50 (2.92)	0.111	14.79 (3.61)	14.49 (3.69)	0.636	12.33 (2.98)	12.18 (3.15)	0.830	3.66 (3.46)	3.04 (3.50)	0.436	10.92 (5.42)	12.46 (6.82)	0.253
	**2-3 hours**	Total	31	41.00 (11.00)	159	40.28 (10.54)	0.731	9.10 (3.09)	8.57 (2.75)	0.337	15.00 (4.55)	14.65 (4.21)	0.680	12.42 (3.05)	12.50 (3.49)	0.908	3.71 (4.37)	4.06 (4.00)	0.664	12.10 (7.18)	11.32 (5.82)	0.515
		Female	19	39.16 (10.73)	82	40.68 (12.05)	0.613	8.16 (2.61)	8.54 (3.12)	0.625	14.47 (4.75)	14.82 (4.80)	0.779	12.26 (2.83)	12.76 (3.93)	0.607	3.37 (4.30)	4.73 (4.49)	0.233	11.53 (7.09)	11.48 (7.09)	0.975
		Male	12	43.92 (11.25)	77	39.86 (8.71)	0.153	10.58 (3.32)	8.60 (2.31)	**0.011**	15.83 (4.26)	14.48 (3.50)	0.230	12.67 (3.50)	12.22 (2.96)	0.637	4.25 (4,62)	3.34 (3.27)	0.399	13.00 (7,54)	11.16 (5.62)	0.317
	**> 3 hours**	Total	4	41.50 (10.02)	186	40.38 (10.62)	0.834	10.50 (3.11)	8.61 (2.80)	0.184	13.00 (1.83)	14.74 (4.29)	0.418	12.75 (3.40)	12.48 (3.43)	0.876	2.75 (2.22)	4.03 (4.08)	0.534	12.25 (5.85)	11.43 (6.06)	0.789
		Female	2	43.00 (7.07)	99	40.34 (11.87)	0.754	10.50 (2.12)	8.42 (3.03)	0.339	13.50 (2.12)	14.78 (4.81)	0.710	13.50 (0.71)	12.65 (3.78)	0.751	4.00 (2.83)	4.48 (4.51)	0.880	15.00 (5.66)	11.41 (6.22)	0.421
		Male	2	40.00 (15.56)	87	40.41 (9.07)	0.950	10.50 (4.95)	8.83 (2.50)	0.360	12.50 (2.12)	14.71 (3.64)	0.396	12.00 (5.66)	12.29 (2.99)	0.895	1.50 (0.71)	3.51 (3.49)	0.421	9.50 (6.36)	11.45 (5.92)	0.647
**Type of supplement**	**Proteins**	Total	22	43.77 (9.11)	168	39.96 (10.71)	0.112	9.82 (2.22)	8.50 (2.85)	**0.038**	15.86 (4.18)	14.56 (4.26)	0.177	13.14 (3.08)	12.40 (3.46)	0.343	4.18 (3.95)	3.98 (4.10)	0.824	13.64 (5,85)	11.16 (6.03)	0.071
		Female	6	45.67 (13.35)	95	40.06 (11.67)	0.260	9.17 (3.66)	8.42 (3.00)	0.561	16.17 (5.49)	14.66 (4.74)	0.457	15.00 (3.35)	12.52 (3.73)	0.115	5,83 (5.85)	4.39 (4.39)	0.446	13.50 (7.71)	11.36 (6.12)	0.414
		Male	16	43.06 (7.41)	73	39.82 (9.40)	0.200	10.06 (1.48)	8.60 (2.65)	**0.004**	15.75 (3.79)	14.42 (3.56)	0.186	12.44 (2.76)	12.25 (3.09)	0.820	3.56 (2.99)	3.44 (3.58)	0.898	13.69 (5.30)	10.90 (5.94)	0.088
	**Vitamins**	Total	8	52.25 (8.48)	182	39.88 (10.38)	**0.001**	12.50 (2.25)	8.48 (2.71)	**<0.001**	19.00 (3.46)	14.52 (4.20)	**0.003**	15.00 (2.73)	12.37 (3.41)	**0.033**	4.88 (3.23)	3.96 (4.09)	0.534	16.38 (5.18)	11.23 (6.00)	**0.018**
		Female	6	51.50 (9.01)	95	39.69 (11.61)	**0.016**	12.33 (2.58)	8.22 (2.89)	**<0.001**	18.33 (3.77)	14.53 (4.75)	0.058	15.17 (2.79)	12.50 (3.75)	0.091	5.33 (3.56)	4.42 (4.53)	0.630	16.83 (5.49)	11.15 (6.11)	**0.029**
		Male	2	54.50 (9.19)	87	40.08 (8.91)	**0.026**	13.00 (2.83)	8.77 (2.47)	**0.019**	21.00 (1.41)	14.52 (3.52)	**0.011**	14.50 (3.54)	12.23 (3.01)	0.296	3.50 (2.12)	3.46 (3.50)	0.987	15.00 (5.66)	11.32 (5.90)	0.386
	**Proteins and vitamins**	Total	2	51.50 (0.71)	188	40.28 (10.58)	0.136	12.50 (2.12)	8.61 (2.79)	0.051	19.00 (1.41)	14.66 (4.25)	0.153	15.50 (0.71)	12.45 (3.42)	0.211	7.00 (5.66)	3.97 (4.04)	0.294	15.50 (0.71)	11.40 (6.06)	0.342
		Female	0		101	40.40 (11.77)			8.46 (3.03)			14.75 (4.77)			12.66 (3.74)			4.48 (4.47)			11.49 (6.20)	
		Male	2	51.50 (0.71)	87	40.15 (9.10)	0.082	12.50 (2.12)	8.78 (2.50)	**0.040**	19.00 (1.41)	14.56 (3.59)	**0.043**	15.50 (0.71)	12.21 (3.01)	0.128	7.00 (5.66)	3.38 (3.41)	0.145	15.50 (0.71)	11.31 (5.93)	0.324
	**Other supplements**	Total	7	40.71 (5.88)	183	40.39 (10.74)	0.936	8,71 (2.29)	8.65 (2.83)	0.953	15.86 (2.79)	14.67 (4.30)	0.469	11.71 (1.38)	12.51 (3.47)	0.202	4.71 (2.36)	3.97 (4.10)	0.636	13.43 (4.50)	11.37 (6.09)	0.378
		Female	5	41.00 (5.20)	96	40.36 (12.03)	0.907	9.00 (2.35)	8.44 (3.06)	0.687	15.20 (1.92)	14.73 (4.88)	0.831	11.80 (1.64)	12.71 (3.82)	0.599	5.20 (1.10)	4.44 (4.58)	0.279	14.60 (4.62)	11.32 (6.26)	0.251
		Male	2	40.00 (9.90)	87	40.41 (9.17)	0.950	8.00 (2.83)	8,88 (2.55)	0.629	14.60 (3.59)	14.59 (3.59)	0.264	11.50 (0.71)	12.30 (3.05)	0.714	3.50 (4.95)	3.46 (3.46)	0.987	10.50 (3.54)	11.43 (5.96)	0.828
**Participation in official competitions**		Total	116	38.59 (10.71)	74	43.23 (9.80)	0.950	8.28 (2.95)	9.24 (2.48)	0.629	14.04 (4.29)	15.76 (4.02)	0.264	11.94 (3.31)	13.34 (3.44)	0.714	3.68 (4.23)	4.50 (3.73)	0.987	10.12 (6.26)	13.53 (5.07)	0.828

Female	70	37.83 (11.19)	31	46.19 (11.14)	**<0.001**	7.93 (2.94)	9.68 (2.91)	**0.007**	13.80 (4.62)	16.90 (4.46)	**0.002**	11.84 (3.42)	14.51 (3.83)	**<0.001**	3.90 (4.58)	5.77 (3.98)	0.051	9.99 (6.09)	14.87 (5.08)	**<0.001**
Male	46	39.76 (9.96)	43	41.09 (8.19)	0.494	8.80 (2.92)	8.93 (2.10)	0.817	14.41 (3.74)	14.93 (3.50)	0.503	12.09 (3.16)	12.49 (2.88)	0.534	3.35 (3.65)	3.58 (3.29)	0.753	10.33 (6.57)	12.56 (4.90)	0.072

M: Media; SD: Standard Deviation; EHQ: Eating Habits Questionnaire; OrNe: Orthorexia nervosa; HeOr: Healthy Orthorexia. Categorical variable with more than two categories were dichotomized. Bold p-values indicate significant differences between the category with a YES response versus all other categories of the variable.
