# Supplementary material for: Orthorexia Profiles in Athletes: A Multidimensional Analysis Using the Eating Habits Questionnaire (EHQ) and the Teruel Orthorexia Scale (TOS)
Source: Nutrients. 2025 Dec 5;17(24):3814. doi: 10.3390/nu17243814 (PMC12735428; doi:10.3390/nu17243814)
Supplement: Supplementary file 1 [file nutrients-17-03814-s001.zip › nutrients-3989902-supplementary/Supplementary Table S2. Descriptive characteristics of the clusters.pdf]

Supplementary Table S2: Descriptive characteristics of the clusters.

		Cluster 1: intermediate profile		Cluster 2: Non-orthorexic profile		Cluster 3: High Orthorexic Profile		
	N	% or M(SD)	N	% or M(SD)	N	% or M(SD)	p	ES
**Age**	83	23.52 (5.12)	75	21.60 (4.53)	32	25.87 (5.29)	**<0.001**	
**Sex** Female	43	51.8	40	53.3	18	56.3	0.912	
Male	40	48.2	35	46.7	14	43.8		
**Diet type**							0.768	0.132
Mediterranean	76	91.6	69	92.0	27	84.4	0.426	0.095
Vegetarian	3	3.6	2	2.7	3	9.4	0.268	0.118
Hyperproteic	3	3.6	3	4.0	1	3.1	0.975	0.016
Others/no specific	1	1.2	1	1.3	1	3.1	0.742	0.056
**Type of sport**							**<0.001**	**0.353**
Running	8	9.6	1	1.3	0	0	**0.019**	**0.204**
Handball	20	24.1	48	64.0	13	40.6	**<0.001**	**0.368**
Cycling	1	1.2	0	0	2	6.3	0.056	0.174
Fitness	33	39.8	7	9.3	8	25.0	**<0.001**	**0.319**
Soccer	10	12.0	10	13.3	1	3.1	0.283	0.115
Others	11	13.3	9	12.0	8	25.0	0.194	0.131
**Frequency of weekly training sessions**							0.061	0.154
1 or 2	9	10.8	9	12.0	4	12.5	0.959	0.021
3 or 4	42	50.6	53	70.7	18	56.3	**0.034**	**0.188**
≥ 5	32	38.6	13	17.3	10	31.3	**0.013**	**0.214**
**Training duration**							0.175	0.154
1-2 hours	50	60.2	56	74.7	23	71.9	0.133	0.146
2-3 hours	13	15.7	11	14.7	7	21.9	0.638	0.069
30-60 minutes	17	20.5	7	9.3	2	6.3	0.051	0.177
> 3 hours	3	3.6	1	1.3	0	0	0.402	0.098
**Participation in official competitions** Yes	39	47.0	60	80.0	17	53.1	**<0.001**	**0.317**
No	44	53.0	15	20.0	15	46.9		
**Consumption of supplements** Yes	22	26.5	7	9.3	11	34.4	**0.004**	**0.242**
No	61	73.5	68	90.7	21	65.6		
**Type of supplement**							0.564	0.249
Proteins	11	13.3	5	6.7	6	18.8	0.165	0.138
Vitamins	2	4.8	1	1.3	1	1.3	0.155	0.140
Proteins and vitamins	1	1.2	1	1.3	0	0	0.344	0.106
Other supplements	6	7.2	7	9.3	1	1.3	0.183	0.168
**EHQ Total Score**	83	42.94 (5.91)	75	31.08 (5.60)	32	55.66 (6.96)	**<0.001**	**0.684**
Behaviors	83	15.41 (2.91)	75	11.35 (2.23)	32	20.78 (3.10)	**<0.001**	**0.604**
Knowledges	83	9.23 (2.13)	75	6.53 (1.62)	32	12.13 (2.37)	**<0.001**	**0.503**
Feelings	83	13.35 (2.10)	75	9.68 (2.33)	32	16.81 (2.51)	**<0.001**	**0.566**
**TOS** OrNe	83	3.95 (2.33)	75	1.16 (1.41)	32	10.78 (3.74)	**<0.001**	**0.669**
HeOr	83	14.07 (3.72)	75	5.69 (3.29)	32	18.13 (3.98)	**<0.001**	**0.649**

M: Media; SD: Standard Deviation; EHQ: Eating Habits Questionnaire; OrNe: Orthorexia nervosa; HeOr: Healthy Orthorexia. Effect sizes (ES) are shown as Cramer's V, except for the scores of the EHQ, TOS questionnaires and their subscales, which are shown as η^2p. The p-value indicates differences between clusters. In bold: statistically significant relationships.
