# Supplementary material for: Orthorexia Profiles in Athletes: A Multidimensional Analysis Using the Eating Habits Questionnaire (EHQ) and the Teruel Orthorexia Scale (TOS)
Source: Nutrients. 2025 Dec 5;17(24):3814. doi: 10.3390/nu17243814 (PMC12735428; doi:10.3390/nu17243814)
Supplement: Supplementary file 1 [file nutrients-17-03814-s001.zip › nutrients-3989902-supplementary/Supplementary Table S3. Cluster-specific partial correlation coefficients (r) among EHQ total and subscale scores, OrNe, and HeOr, adjusted for sex, age, and BMI..pdf]

Supplementary Table S3. Cluster-specific partial correlation coefficients (r) among EHQ total and subscale scores, OrNe, and HeOr, adjusted for sex, age, and BMI.

		EHQ Total Score	Knowledges	Behaviors	Feelings	OrNe
**Knowledges**	Cluster 1: Intermediate profile	**0.663****				
	Cluster 2: Non-orthorexic profile	**0.706****				
	Cluster 3: High orthorexic profile	**0.719****				
**Behaviors**	Cluster 1: Intermediate profile	**0.832****	**0.444****			
	Cluster 2: Non-orthorexic profile	**0.841****	**0.603****			
	Cluster 3: High orthorexic profile	**0.896****	**0.475****			
**Feelings**	Cluster 1: Intermediate profile	**0.710****	0.177	**0.390****		
	Cluster 2: Non-orthorexic profile	**0.827****	**0.328****	**0.532****		
	Cluster 3: High orthorexic profile	**0.808****	0.342	**0.667****		
**OrNe**	Cluster 1: Intermediate profile	0.081	-0.019	0.122	0.056	
	Cluster 2: Non-orthorexic profile	**0.273***	0.011	0.029	**0.335****	
	Cluster 3: High orthorexic profile	-0.032	0.055	-0.067	-0.004	
**HeOr**	Cluster 1: Intermediate profile	0.124	-0.019	-0.030	0.185	**-0.223***
	Cluster 2: Non-orthorexic profile	**0.610****	**0.333****	**0.555****	**0.504****	0.060
	Cluster 3: High orthorexic profile	**0.388***	0.275	**0.453***	0.190	-0.015

EHQ: Eating Habits Questionnaire; OrNe: Orthorexia nervosa; HeOr: Healthy Orthorexia.

**. The correlation is significant at the 0.01 level (bilateral).

*. The correlation is significant at the 0.05 level (bilateral).

In bold: statistically significant relationships.
